# Sedentary behaviour and physical activity levels in Swedish adolescents with and without intellectual disabilities

**DOI:** 10.1186/s12887-026-06679-9

**Published:** 2026-03-05

**Authors:** Marie Lund Ohlsson, Craig A. Staunton, Eva Flygare Wallén, Erik P. Andersson, Sanna Fjellström

**Affiliations:** 1 Department of Health Sciences, Swedish Winter Sport Research Centre, Mid Sweden University, Östersund, Sweden; 2 Department of Physiology, Nutrition and Biomechanics, The Swedish School of Health and Sport Sciences (GIH), Stockholm, Sweden; 3 Department of Environmental and Bioscience, School of Business, Innovation and Sustainability, Halmstad University, Halmstad, Sweden; 4 Department of Neurobiology, Care Sciences and Society (NVS), Division of Occupational Therapy, Karolinska Institutet (KI), Stockholm, Sweden; 5 Health and Social Care Administration, Municipality of Östersund, Östersund, Sweden; 6 Department of Living Conditions and Lifestyle, The Public Health Agency of Sweden, Östersund, Sweden

**Keywords:** Body composition, Exercise, Health, Intellectual impairment, Youth

## Abstract

**Background:**

Physical activity is essential for health and well-being during adolescence, and active behaviour early in life predicts higher physical activity levels in adulthood. Although adolescents with intellectual disability (ID) consistently show lower activity levels than peers without ID, national environments—such as school structures, disability support systems, and access to inclusive leisure activities—may influence these patterns. There is limited evidence from Sweden, a country with distinct educational and support frameworks for youth with ID. The present study aimed to examine physical activity patterns among Swedish adolescents with and without ID using accelerometer data.

**Methods:**

Physical activity was measured objectively using hip-worn accelerometers (ActiGraph GT3X) over seven consecutive days. This cross-sectional study included 45 adolescents with mild-to-moderate ID (median [IQR], 17.0 [14.0–19.0] years; 45.2% females) and 70 adolescents without ID (16.0 [15.0-16.3] years; 62.2% females). Physical activity was categorised as sedentary behaviour (SB), light physical activity (LPA), and moderate-to-vigorous physical activity (MVPA) and analysed across school days, weekend days, and separately for daytime and evening periods on school days.

**Results:**

Overall, the relative amount of SB was similar between groups (*p* > 0.05), but significant differences were observed for LPA and MVPA. Adolescents with ID accumulated more LPA during school-day daytime hours (23% vs. 19%; *p* = 0.012), while peers without ID accumulated more MVPA during leisure time, such as school-day evenings (*p* = 0.025) and weekends (*p* = 0.039). For both groups, MVPA was higher on school days than on weekends (*p* < 0.001). Among adolescents with ID, SB increased markedly on weekends (72% vs. 77%; *p* < 0.001).

**Conclusion:**

Adolescents with ID were generally less physically active than peers without ID, except during school-day daytime, where the MVPA was similar and LPA was higher. Leisure time, particularly weekends and school-day evenings, seems to be a critical period in achieving sufficient MVPA among adolescents with ID. Targeted interventions and coordinated support from key stakeholders such as school health services, paediatric health care, social care services and organised sports, with a particular focus on unstructured time, may help promote active lifestyles and reduce health disparities in this population.

**Supplementary Information:**

The online version contains supplementary material available at 10.1186/s12887-026-06679-9.

## Introduction

Physical activity is essential for health and well-being during adolescence, as it contributes to physical, mental, and social development. Despite these well-established benefits, many adolescents fail to meet recommended levels of physical activity, and sedentary behaviour (SB) occupies a substantial portion of their daily lives [1]. Adolescents with intellectual disabilities (ID), defined by significant limitations in intellectual functioning (IQ < 70) and adaptive behaviour, are particularly vulnerable to inactivity and its consequences [2, 3]. This population faces substantial health challenges, as approximately 45% are overweight and 20% live with obesity. These rates are about 50% higher than those of peers without ID, placing them at increased risk for cardiovascular disease and type 2 diabetes [4–7]. Inactivity is also associated with a poorer quality of life, and individuals with ID generally report a lower quality of life than those without ID [8].

Recognizing these risks, the World Health Organization updated its guidelines for physical activity in 2020 to explicitly include people with disabilities [9]. For children and adolescents aged 5–17 years, the recommendations continue to emphasise at least 60 min of moderate-to-vigorous physical activity (MVPA) per day, along with vigorous-intensity and muscle-strengthening activities performed at least three times per week. Establishing active behaviours early in life is critical, as physical activity in childhood predicts higher activity levels in adulthood [10, 11].

Despite this, research consistently shows that adolescents with ID are less active than peers without disabilities. Studies from Iceland, the United States, and the Netherlands report that adolescents with ID rarely meet physical activity recommendations and exhibit strikingly low fitness levels [12–14]. In Nordic and European contexts, including Sweden, research remains limited. A previous study by Sundahl et al. [15] found that female adolescents with ID took fewer steps than peers without ID, with activity levels tending to decline on weekends. Other studies highlight motivational factors and social barriers, showing that adolescents with mild ID often fear exclusion and rely on support from significant others to participate in physical activity [16, 17]. Given that adolescents’ physical activity levels are context dependent and can vary across countries [18] and socio-economic conditions [19], adolescents without ID from the same schools were included as a reference group to allow for contextually grounded comparisons.

In Sweden, structured school days should provide adapted support that facilitates participation in physical activities according to the curriculum [20]. In contrast, participation in leisure time activities for adolescents with ID is more dependent on external support, such as assistance from family members or staff, as well as the availability of suitable activities [21]. The reduced structure during leisure time may therefore contribute to lower physical activity levels. Leisure time is particularly important for promoting physical activity and psychosocial well-being, as engagement in social activities during free time positively influence self-esteem, mental health, and physical fitness [22, 23]. Despite these benefits, adolescents with ID participate less in leisure and social activities than their typically developing peers [8, 17, 24]. Also, among adolescents without ID, the levels of MVPA during leisure periods are lower [25]; however, MVPA during these periods appears to be a key protective factor against the development of overweight and obesity [26].

Accelerometer-based measurements provide an objective method for capturing physical activity patterns and overcoming the limitations of self-report [27]. As such, this measurement tool is particularly well-suited to assessing how physical activity differs between adolescents with and without ID across school days, weekends, and evenings, or how these patterns relate to body composition. Addressing this gap is essential for developing targeted interventions that promote active lifestyles among adolescents with ID.

The aim of this study was to examine physical activity patterns in Swedish adolescents aged 11–20 years with and without ID using accelerometer data and assess the associations between physical activity patterns and body composition. We hypothesised that adolescents with ID would engage in less MVPA and spend more time sedentary, particularly during leisure periods, compared to adolescents without ID.

## Methods

### Overview

This study was conducted in small cities (20 000–100 000 inhabitants) in central Sweden, with regions classified as rural (4.5 inhabitants/km^2^). A total of 122 adolescents with mild to moderate ID and 287 adolescents without ID were invited to participate in the study. Of these, 135 adolescents volunteered to participate in the study, and 115 were included in the final analyses that comprised of 45 with ID (85% of the initial volunteers) and 70 without ID (85% of the initial volunteers). This cross-sectional study involved a quantitative assessment of physical activity levels using a hip-worn accelerometer over a 7-day period, along with measurements of body composition. To be eligible, participants had to be able to walk independently without the use of assistive devices (i.e., walking aids, such as crutches or walkers). The study was pre-approved by the Swedish Ethical Review Authority (Dnr 2022-06757-01) and conducted in accordance with the declaration of Helsinki. Clinical trial number: not applicable.

### Participants

Schools serving grades 5 to 9 (11 to 16 years old) of compulsory education and upper secondary schools (17–20 years old) in central Sweden were invited to participate during 2023 and 2024. In Sweden, upper secondary school for adolescents with ID spans four years (up to 20 years old) while for adolescents without ID the span is three years (up to 19 years old). This justified the inclusion of participants up to an age of 20 years. Information about the study was distributed to six schools, comprising four compulsory schools and two upper secondary schools, which serve students with ID in the region. Five schools agreed to participate. Additionally, three of these schools, which also included classes with students without ID, were invited to contribute as a reference group. In consultation with school principals and teachers, it was decided that only students in classes for adolescents with milder ID would be offered to participate. In Sweden, students with ID most often go to the same schools as students without ID. However, the students with ID follow a different curriculum from students without ID, and most often they go into separate classes. There are two curricula for students with ID, roughly described as one for students with milder ID and one including a broader topic adapted for students with more severe ID. There is no strict definition linking the level of ID to a specific type of curriculum; instead, it is determined by the level of support that the student requires [20]. The selection of classes, including those with students without ID, was made in consultation with the principals and teachers.

First, information was sent out to all caregivers and students and the teachers introduced the study to their classes. Secondly, the researchers provided an in-depth explanation of the study to the students, either in person or digitally. During these sessions, the students and teachers had the opportunity to ask questions. According to the Swedish Ethical Review Authority, participants aged 15 years and older provided their own consent, with caregivers being informed via email. For participants under 15 years, consent was provided by both caregivers (or one caregiver if there was only one) to confirm that their child was willing and able to participate.

### Outcomes

Physical activity levels were measured using the hip-worn ActiGraph GT3X monitor (Ametris (formerly 145 Actigraph), Pensacola, USA), an accelerometer with established reliability and validity for assessing activity and rest [28–30]. The monitor was placed on the right hip for seven consecutive days during waking hours, except during water-based activities. Each morning, a text message reminder was sent to participants and/or caregivers (according to their preference), encouraging them to put the monitor back on after sleep.

Stature was measured to the nearest 0.1 cm using a manual stadiometer. Body composition was measured using a Body Composition Analyser (InBody 270, Seoul, South Korea) based on Bioelectrical Impedance Analysis (2016 InBody Co., Ltd). Electrodes were cleaned before each assessment, and measurements were repeated in accordance with the device’s validation requirements. Participants wore light clothing (e.g., a single layer, such as a T-shirt and tights), stood barefoot with their feet aligned on the foot electrodes, and held the hand electrodes with their thumbs placed on the oval pads and their arms extended slightly from their bodies. Skeletal muscle mass and fat mass were measured in kilograms. Participants received no instructions concerning the intake of food and drinks before the measurement.

### Data analysis

The accelerometers sampled data at 30 Hz, and counts were recorded in 15-second epochs. Data were included in the analysis if the monitor was worn for at least 500 min per day on a minimum of three school days (weekdays) and a minimum of one weekend day. Accelerometer data were processed in ActiLife, ActiGraph’s software (ActiGraph LLC, version 6.13.4). The device was not worn during sleep at night, taken off at bedtime, and put back on at wake-up time. The detection of non-wear time was determined by the Choi wear time classification algorithm [31]. Total physical activity was estimated by summing all activity counts recorded on the vertical axis throughout the monitoring period. This total was then divided by the number of wear time minutes during the specific period, resulting in an average activity level expressed as counts per minute. To estimate time spent in different activity intensities, the following cut-points were applied; SB = 0–100 counts·minute^− 1^, LPA = 101–2295 counts/minute, and MVPA ≥ 2296 counts/minute These thresholds were based on a validation study that demonstrated good agreement between accelerometer results and measures of oxygen uptake (i.e., indirect calorimetry) and heart rate [32].

Outcome variables included minutes per day spent in SB, LPA, and MVPA. These variables were computed as mean values within the following time domains: total (average over all included days), school days (weekday average), weekend (average of weekend days). For school days, two additional sub-periods were also included: school-day daytime (wake-up time to 16:00) and school-day evening (16:00 to bedtime).

Normality was assessed using Shapiro–Wilk tests, supported by visual inspection of histograms and Q–Q plots. These assessments indicated that variables showed deviations from a normal distribution. As such, descriptive statistics were calculated and are presented as medians and interquartile ranges (IQR) and non-parametric statistical methods were applied. Spearman’s rank correlation coefficient (ρ) was used to assess the correlation between wear time and various activity levels (SB, LPA, and MVPA). Significant correlations were found across all time domains (i.e., for: total, school days, weekends, and school-day evenings), except for school-day daytime, where no significant correlation was observed. Consequently, both absolute and relative (percentage of wear time) values of SB, LPA, and MVPA were analysed.

Between-group differences (i.e., ID vs. non-ID) were assessed using the Mann–Whitney U test. Within-group comparisons were conducted using the Wilcoxon Signed Rank test. A *p*-value of *<* 0.05 was considered statistically significant. Effect sizes were calculated using the rank-biserial correlation, r(rb), derived by dividing the *Z*-value by the square root of *N*, and interpreted according to Cohen’s guidelines for correlations with definitions for small (*r* = 0.10–0.29), medium (*r* = 0.30–0.49), and large (*r* ≥ 0.50) [33].

## Results

### Participant characteristics

A total of 45 participants with ID and 70 participants without ID reached the wear time criteria of the accelerometer use and were included in analyses. The median age was 17.0 years in the ID group and 16.0 years in the non-ID group, and 45.2% and 62.2% were females, respectively. The average accelerometer wear-time was 824.1 (770.1–884.6) and 867.3 [812.3-938.2] minutes per day in the ID and non-ID groups, respectively (*p* = 0.010). An overview of the participant characteristics is presented in Table 1.

**Table 1:** Participant characteristics in median [IQR] for all adolescents and for the groups with and without ID. Differences between the ID and non-ID groups are presented with *p*-values and effect sizes (rank-biserial correlations), with significant differences marked in bold

	All (*n* = 115)	ID (*n* = 45)	Non-ID (*n* = 70)	*p*-value	Effect size
Age (years)	16.0 [15.0–17.0]	16.0 [14.0–19.0]	16.0 [15.0–16.3]	0.283	0.10
Stature (cm)	169.5 [160.5–174.8]	169.5 [158.8–174.5]	169.5 [161.8–175.0]	0.801	0.02
Body mass (kg)	62.0 [52.7–70.1]	64.0 [54.4–81.7]	60.6 [52.5–67.8]	**0.045**	**0.19**
BMI (kg/m^2^)	21.1 [19.3–24.3]	22.8 [19.1–28.4]	20.9 [19.3–23.1]	**0.028**	**0.21**
SMM (kg)	24.3 [21.2–29.7]	25.6 [21.6–30.2]	24.1 [21.0–29.6]	0.584	0.05
FM (kg)	14.5 [9.9–21.1]	16.2 [10.4–30.2]	13.9 [9.5–17.6]	**0.008**	**0.25**

*Abbreviations*: body mass index (*BMI*), skeletal muscle mass (*SMM*), and fat mass (*FM*)

### Physical activity pattern comparisons between adolescents with and without ID

**Fig. 1:**
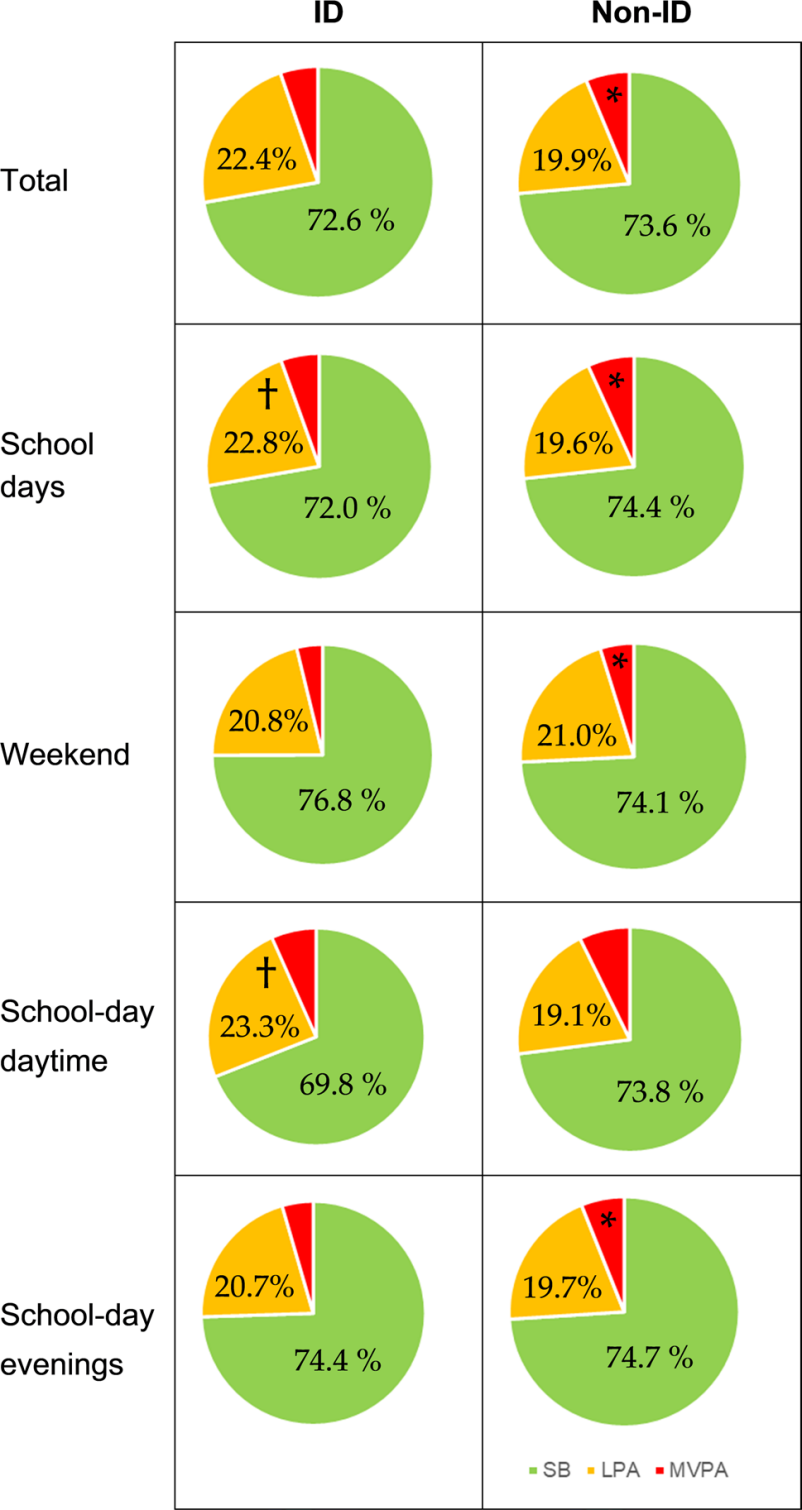
Relative distribution (% of wear time) of sedentary behaviour (SB, in green), low intensity physical activity (LPA, in yellow), and moderate to vigorous physical activity (MVPA, in red) among adolescents with and without ID across different time domains. An asterisk (*) indicates significantly higher MVPA in the non-ID group, and a cross (†) indicates significantly higher LPA for the ID group

The distribution of physical activity intensities (SB, LPA, MVPA) for adolescents with and without ID is presented as the percentage of total wear time (i.e., “total”) for school days, weekends, school-day evenings, and school-day daytime (Fig. 1). Overall, the relative proportion of SB was similar between groups across all time domains (*p* > 0.05), except during school-day daytime, where adolescents without ID were significantly more sedentary than those with ID (*p* = 0.006). Significant group differences were observed for relative LPA during daytime on school days (*p* < 0.001) and across the entire school day (*p* = 0.012), with adolescents with ID spending a greater proportion of wear time in LPA (23.3%) than peers without ID (19.1%). For relative MVPA, participants without ID consistently accumulated a higher proportion of time in MVPA than those with ID, with significant differences on school days (*p* = 0.047), weekends (*p* = 0.039), and school-day evenings (*p* = 0.025), but not during school-day daytime. These findings suggest that while total SB proportions did not differ markedly between groups, LPA and MVPA vary between groups and time domains. Particularly, school-day daytime appears to support more favourable activity attainments for adolescents with ID, while less structured periods, such as weekends and school-day evenings, were associated with lower levels of physical activity.

Descriptive analyses of absolute values are presented in Table 2 and show the absolute differences in physical activity patterns between adolescents with and without ID. Overall, participants without ID accumulated more MVPA than those with ID, with the largest differences observed during weekends and school-day evening periods/time domains. During school-day daytime, however, the groups showed similar MVPA levels, while adolescents with ID accumulated more LPA (medium effect size r(rb) = 0.31), which was consistent with the relative analysis.

**Table 2:** Sedentary behaviour (SB), low intensity physical activity (LPA), and moderate to vigorous physical activity (MVPA) in minutes (median [IQR]) for adolescents with and without intellectual disability (ID) in five different time domains (i.e., total, school days, weekend, school-day daytime wake-up time-16:00, and school-day evenings, 16.00 – bedtime). Between-group differences are presented with *p*-values and effect sizes (rank-biserial correlations, r(rb)), with significant differences marked in bold

	All (*n* = 115)	ID (*n* = 45)	Non-ID (*n* = 70)	*p*	Effect size(*r*)
Total					
SB	628.5 [539.4–687.8]	583.6 [519.7–665.2]	645.5 [569.6–692.3]	**0.012**	**0.23**
LPA	171.0 [145.3–213.5]	177.5 [151.7–213.0]	166.7 [141.5–215.0]	0.362	0.09
MVPA	47.0 [34.9–60.3]	44.1 [25.7–55.4]	53.3 [38.7–61.2]	**0.011**	**0.24**
School days					
SB	641.8 [557.0–704.4]	604.7 [527.8–687.6]	647.9 [573.1–711.8]	**0.047**	**0.19**
LPA	179.0 [148.6–212.3]	191.7 [157.2–222.8]	165.5 [145.5–205.3]	**0.041**	**0.19**
MVPA	54.7 [40.2–67.2]	46.2 [32.5–63.3]	57.3 [44.2–71.2]	**0.015**	**0.23**
Weekend					
SB	570.5 [499.8–653.5]	546.9 [492.8–610.5]	597.8 [509.0–673.1]	**0.024**	**0.21**
LPA	161.8 [112.0–211.3]	153.6 [105.2–185.9]	166.1 [130.3–218.5]	0.155	0.13
MVPA	27.5 [13.5–48.8]	17.8 [9.3–46.0]	31.1 [20.6–56.4]	**0.010**	**0.24**
School-day daytime					
SB	367.6 [335.3–401.2]	354.5 [324.7–394.9]	372.5 [342.1–407.6]	**0.028**	**0.21**
LPA	105.4 [80.4–133.7]	119.1 [96.8–150.7]	93.9 [76.3–123.8]	**< 0.001**	**0.31**
MVPA	36.8 [24.9–44.6]	33.9 [19.1–47.6]	37.4 [31.4–44.5]	0.162	0.13
School-day evenings					
SB	261.3 [210.2–319.4]	254.8 [198.7–301.5]	278.6 [229.1–339.0]	0.073	0.17
LPA	72.9 [54.6–91.2]	65.7 [50.5–91.2]	73.0 [57.1–91.3]	0.313	0.09
MVPA	16.2 [9.3–26.5]	13.7 [6.5–19.8]	17.5 [11.9–31.2]	**0.004**	**0.27**

### Physical activity patterns within groups

For adolescents with ID, MVPA was significantly higher on school days compared to weekends (*p* < 0.001), while SB was significantly higher on weekends than on school days (*p* < 0.001). For adolescents with ID, descriptive statistics showed that relative SB on school days averaged 72.0% [68.0–76.2], increasing to 76.8% [68.7–82.5] on weekends. These patterns suggest that, for the ID group, activity levels fluctuate substantially across the week, with weekends characterised by lower MVPA and higher SB.

For adolescents without ID, a similar pattern of higher MVPA on school days compared to weekends was observed (*p* < 0.001). However, unlike the ID-group, no significant differences were observed in SB between school days and weekends, in either relative or absolute values.

### Body composition, age, and physical activity patterns

Participants without ID had significantly lower body mass, BMI and fat mass, compared to those with ID with small effect size (Table 1). In the non-ID group, MVPA was negatively correlated with fat mass, i.e., higher levels of MVPA were associated with lower fat mass; however, this association was not observed in the ID group. Additionally, age was negatively correlated with MVPA in the non-ID group, whereas no such association was found among adolescents with ID.

## Discussion

The aim of this study was to examine physical activity patterns in Swedish adolescents with and without ID and to assess the associations between physical activity patterns and body composition. This study identified important differences in physical activity patterns between adolescents with ID and without ID. Notably, MVPA during school-day daytime was similar for both groups, whereas adolescents with ID spent a greater proportion of time in LPA (23.3% vs. 19.1%). In contrast, leisure-time MVPA was markedly lower among adolescents with ID, with significant differences observed on school days, weekends, and school-day evenings. These results suggest that unstructured periods such as school-day evenings and weekends represent a critical barrier to physical activity for adolescents with ID, whereas during school time, a structured environment, appears to facilitate more consistent engagement in physical activity. This interpretation reinforces previous research from Sweden [15] and the United States [13], which also found higher activity levels on weekdays than weekends among adolescents with ID. A recent Swedish study also showed lower physical activity levels for adolescents with other impairments, such as physical, vision, hearing and neuropsychiatric impairments, compared to adolescents without impairments, especially during weekday leisure time together with lower participation in leisure time sport activities [25]. Together, these results emphasise the importance of structured settings in promoting physical activity [26].

Although group differences in total MVPA and SB between were statistically significant, the effect sizes were small to medium, warranting cautious interpretation of the practical implications. Further research, especially from large-scale studies, is needed to better understand these groups differences. Nevertheless, the pronounced school day–weekend contrast within the ID group, where MVPA was significantly higher on school days than weekends, and with SB also being higher on weekends, indicate that targeted interventions during leisure time could be beneficial for health promotion. The higher levels of LPA observed during school-day daytime among adolescents with ID may reflect adaptations within special education settings, such as more frequent breaks and practical, movement-oriented learning activities. Such strategies could be beneficial in all educational contexts, as reducing SB and integrating physical movement into lessons may promote both engagement and health for all students.

Patterns within the groups revealed that adolescents without ID were generally more active during school-day evenings and weekends. In this group, a negative correlation between age and MVPA indicated that older participants tended to be less physically active, which aligns with developmental trends reported in previous research [34]. This age-related decline in MVPA was not present in the ID group, possibly due to structured routines during school hours, where most of the MVPA and LPA occurred. Previous research has indicated that higher adiposity is associated with lower MVPA levels among youth without disability [35], i.e., adolescents with lower fat mass tend to engage more in MVPA. This aligns with the current findings, as demonstrated by the significant negative correlation between body fat percentage and MVPA in the non-ID group, as well as by the higher adiposity and lower MVPA levels observed in the ID-group, compared with their peers without ID. One plausible explanation is that reduced fat mass may facilitate movement and make higher-intensity activities less physically demanding, thereby encouraging greater participation. Future studies should explore whether interventions targeting body composition can indirectly enhance physical activity engagement.

The findings emphasise that leisure time represents a critical context where additional support is needed for adolescents with ID. Limited availability of trained staff and responsible adults during these periods creates barriers to engagement and inclusion [21]. Schools generally provide structured environments that facilitate participation, but leisure settings often lack comparable resources and organizational frameworks. This gap contributes to disparities in activity levels, not only during childhood and adolescence but potentially extending into adulthood, when daily routines may involve even less physical activity. There are multifaceted barriers to participation in physical activity among people with ID, including personal factors (health problems and preferences for sedentary behaviours), social factors (such as limited access to adapted activities and lack of inclusion), environmental factors (social and physical) [36]. In addition, individuals with ID are often more dependent on personal support to engage in physical activity [37, 38]. Self-reported data of Swedish people with disabilities indicate lower levels of physical activity, lower participation in leisure-time and sports activities, as well as lower well-being and socio-economical resources [38].Therefore, promoting equity in leisure-time activity is a complex challenge, as barriers operate across multiple levels, such as individual factors, social support, availability of accessible and adapted activities, transportation and financial resources [39]. Regarding promotion of physical activity, previous research has identified several approaches, including structured programs, environmental modifications, family involvement [40]. In addition, key stakeholders, including school-health services, paediatric health care, social care services, and organised sports, play important roles in promoting healthy lifestyles. Cross-sector collaboration among these stakeholders may therefore help to increase physical activity levels and improve health equity [41, 42].

Addressing these structural gaps is essential to ensure equitable opportunities for physical activity beyond the school environment and to promote health and social participation across the life course. Key components include adapting activities and environments, developing supportive organizational policies, and actively involving individuals with disabilities in the design and implementation of programs [43]. However, evidence regarding the long-term effectiveness of such strategies remains limited [43]. Future research should therefore focus on interventions that promote participation in leisure-time physical activity and examine their sustainability over time. Additionally, longitudinal studies are needed to determine whether such initiatives lead to improved health and social inclusion for individuals with ID.

Many studies emphasise the need for more research on physical activity among people with ID. In this study, the authors realised that including adolescents with ID requires additional time and resources. The recruitment process demands extra effort to ensure that participants fully understand the study procedures and have opportunities to ask questions. Even after obtaining consent, providing instructions and mounting the accelerometers took more time than expected. These experiences highlight that conducting high-quality research with individuals with ID requires greater resources, but such investments are crucial for generating knowledge that can support more active and healthy lives within this population.

### Methodological considerations and future perspectives

To reduce the effect of context-dependent aspects on physical activity levels, the participants without ID were recruited from the same schools as the ID participants. However, the participants with ID lived farther away from their schools as these schools serve larger catchment areas. This reflects a contextual difference, i.e. higher proportion of adolescents with ID in this sample reside in rural areas compared to the non-ID group. Living in rural areas is generally related to lower levels of physical activity [44] and longer time for daily commuting time to school. In addition, the sex distribution differed between groups, with a higher proportion of females in the non-ID group. Previous research has shown that among adolescents without ID, female adolescents tend to engage in less MVPA than males [45]. This sex-related pattern seems not to be present for adolescents with ID; instead, this pattern seems to onsets later in adulthood [46].

Adolescents with more severe ID were not included in the current study, based on recommendations from school staff. As this study was feasible future research should continue to investigate activity patterns and influencing factors to physical activity among people, also with more severe ID, and evaluate strategies designed to overcome barriers specific to this group. However, as individuals with more severe ID also have a higher incidence of other health conditions, such as musculoskeletal impairments, this further contributes to lower levels of physical activity [14, 47]. Nevertheless, evidence suggests that regular physical activity and targeted motor skill interventions can improve health outcomes even among children with severe ID, underscoring the need for inclusive and adaptable approaches across all levels of intellectual disability [40].

A limitation of this study is the absence of data on the exact school-time and whether participants, with or without ID, attended after-school programs before 16:00. This lack of information prevents us from conclusively determining that the highest activity levels occurred exclusively during school hours. Nevertheless, the findings clearly indicate a marked difference compared to evenings and weekends, where activity levels were substantially lower. Previous research suggests that structured after-school programs can significantly contribute to overall physical activity among youth [48], highlighting the need for future studies to account for these contexts.

Careful consideration of measurement and analysis is important when interpreting the findings of the current study. Hip-worn accelerometers provide accurate estimates of intensity but may reduce participant compliance compared to wrist-worn devices [49, 50]. Additionally, accelerometers have known limitations in capturing certain activities, such as cycling, swimming, and resistance training [50].

Differences in the interpretation of SB also highlight the importance of reporting both absolute and relative measures. Although total SB was higher in the non-ID group, no significant difference emerged when SB was expressed as a percentage of wear time, illustrating how variations in wear duration can influence results. Since the data were not normally distributed and non-parametric methods were employed, we were unable to adjust for wear time in the regression models. This limitation implies that variability in wear time may affect absolute estimates of SB, LPA, and MVPA. To mitigate this bias, both absolute values (minutes/day) and relative values (percentage of wear time) were analysed, as recommended in the literature to improve comparability across participants and studies [51–53].

In the present study, we opted to process accelerometer data using the vertical axis (VA) and apply Evenson et al. [32] cut points to classify physical activity intensity. We also opted for a 15-second epoch length due to the adolescent sample rather than the traditional 60-second epochs more suitable for adults [50]. However, both the choice of axis and epoch length inherently influence activity classification. These methodological choices should therefore be considered when comparing our results with studies using different accelerometer processing decisions.

Measuring body composition using Bioelectrical Impedance Analysis comes with its limitations, and it has less accuracy than, for example, a Dual-Energy X-ray Absorptiometry (DXA) or a skin-fold calliper procedure. As this study was performed in schools, the Bioelectrical Impedance Analysis, although with its limitations, was our choice due to the portability, the low time consumption, and the non-invasive/non-touching method for assessing body composition [54].

## Conclusion

Adolescents with ID were generally less physically active than peers without ID, except during school-day daytime, where the MVPA was similar and LPA was higher. Leisure time, particularly during weekends and school-day evenings, appears to be a critical period in achieving sufficient MVPA among adolescents with ID. Targeted interventions and coordinated support from key stakeholders such as school health services, paediatric health care, social care services and organised sports, with a particular focus on unstructured time, may help promote active lifestyles and reduce health disparities in this population.

## Supplementary Information

Supplementary Material 1.

## Data Availability

The meta dataset supporting the conclusions of this article is included as an additional file. While the raw data are available from the corresponding author on reasonable request.

## Abbreviations

ID: intellectual disability
SB: sedentary behaviour
LPA: light physical activity
MVPA: moderate-to-vigorous physical activity
